# Supplementary material for: Individual bat virome analysis reveals co-infection and spillover among bats and virus zoonotic potential
Source: Nat Commun. 2023 Jul 10;14:4079. doi: 10.1038/s41467-023-39835-1 (PMC10333379; doi:10.1038/s41467-023-39835-1)
Supplement: Supplementary file 1 — Supplementary Information [file 41467_2023_39835_MOESM1_ESM.pdf]

Supplementary Information for

“Individual bat viromes reveal the co-infection, spillover and emergence risk of potential zoonotic viruses”

Supplementary Figures

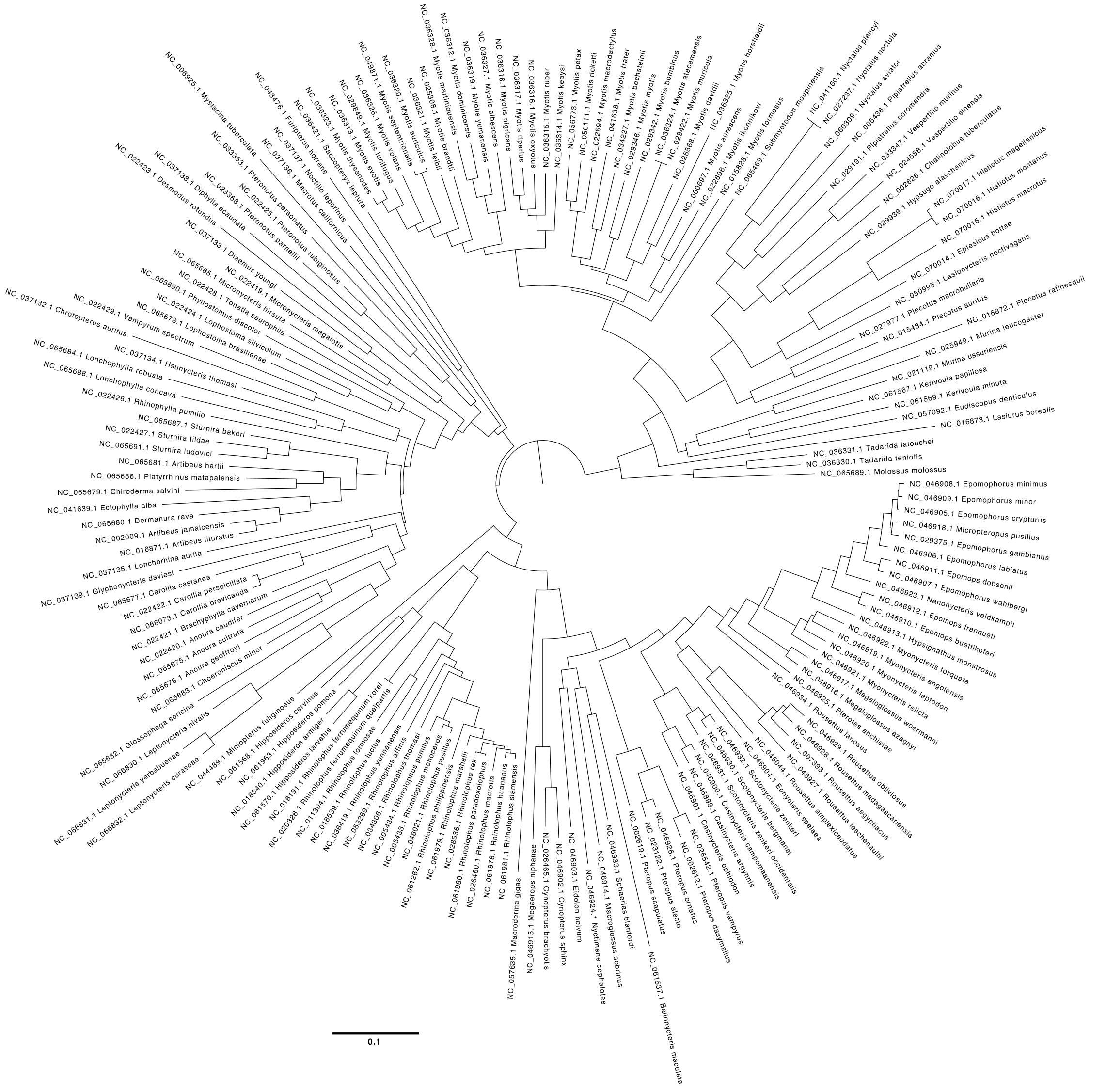

Supplementary Fig. 1 | The Phylogenetic tree of bats. This tree includes all the reference COI sequence from NCBI. The tree was estimated using nucleotide sequences of bat COI gene utilising a maximum likelihood (ML) method.

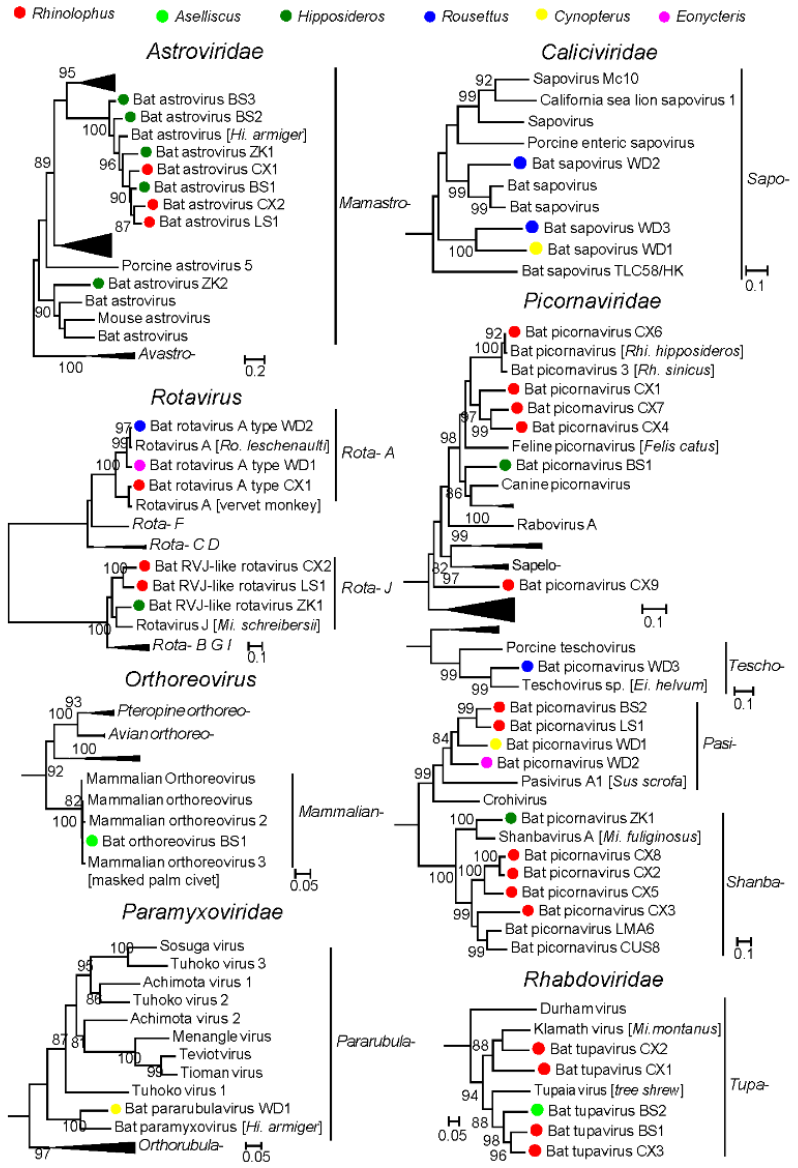

Supplementary Fig. 2 | The evolutionary relationships of the RNA viruses identified in this study. The phylogenetic trees were estimated using a maximum likelihood method based on the RdRp protein. All trees were midpoint-rooted and the branch length indicates number of nucleotide substitutions per site. For clarity, only support values >80% are shown. Dots indicate viruses detected in our samples and colors represent host genus.

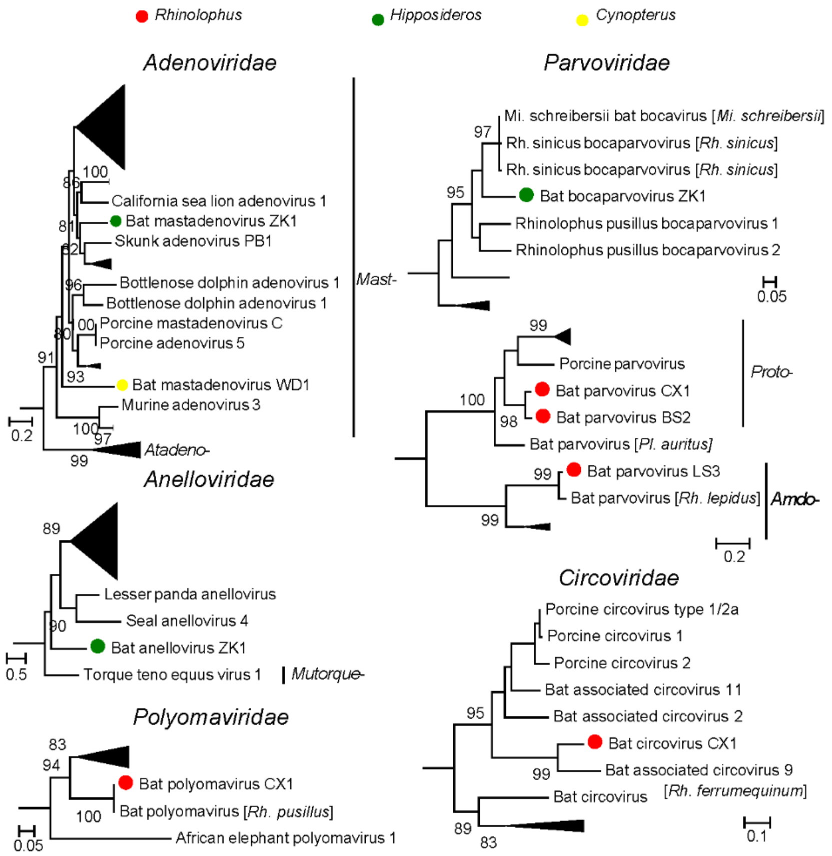

Supplementary Fig. 3 | The evolutionary relationships of the DNA viruses identified in this study. These phylogenetic trees were estimated using a maximum likelihood method based on DNA pol or LTAg (*Polyomaviridae*), ORF1 (*Anelloviridae*), and NS1 (*Parvoviridae*) protein. All trees were midpoint-rooted, and the branch length indicates number of nucleotide substitutions per site. For clarity, only support values >80% were shown. Dots indicate viruses detected in our samples, and colors represent host genus.

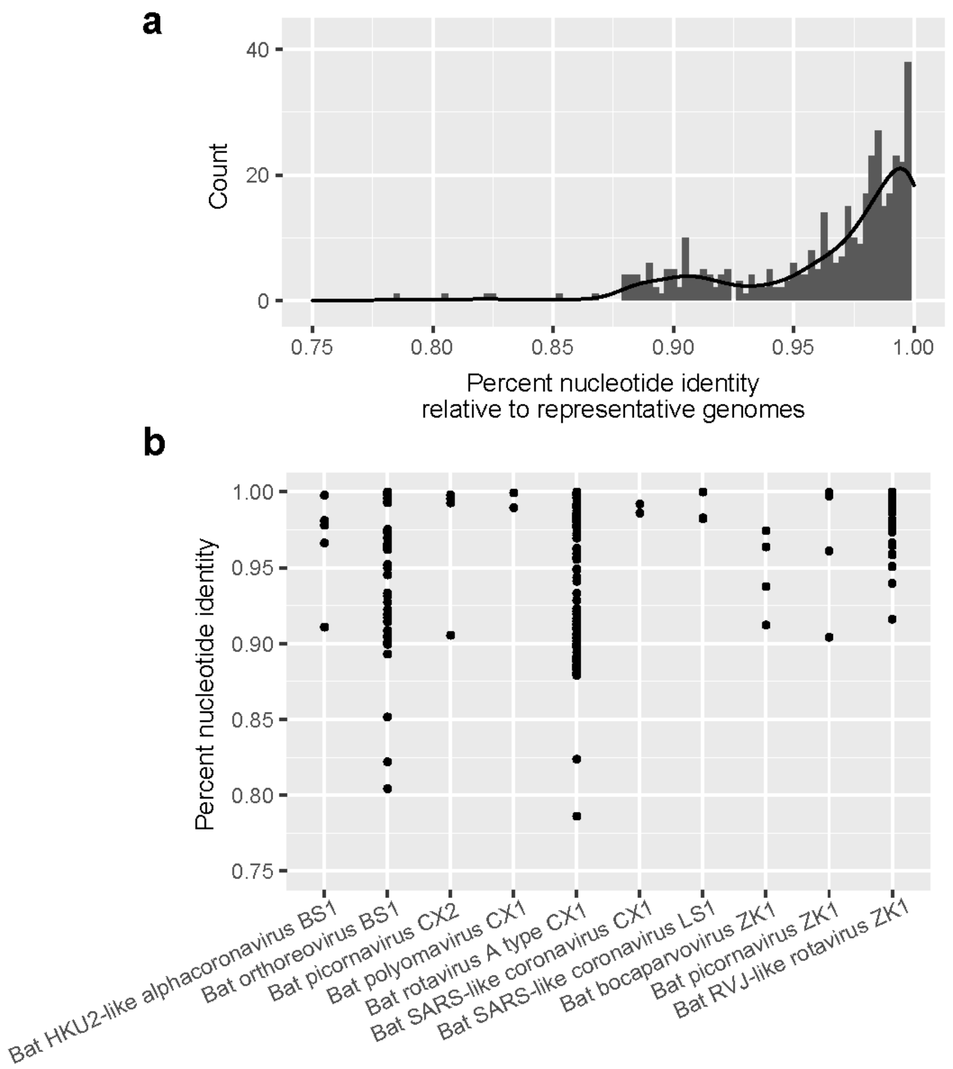

Supplementary Fig. 4 | The whole-genome nucleotide identity of viral strains versus the corresponding representative genomes. (**A**) Histogram of percentage genome identity to representative genomes. All viral species and all strains were shown. (**B**) Percentage genome identity to representative genomes. This plot displayed the 10 viral species that were detected among different bat species (cross-species transmission).

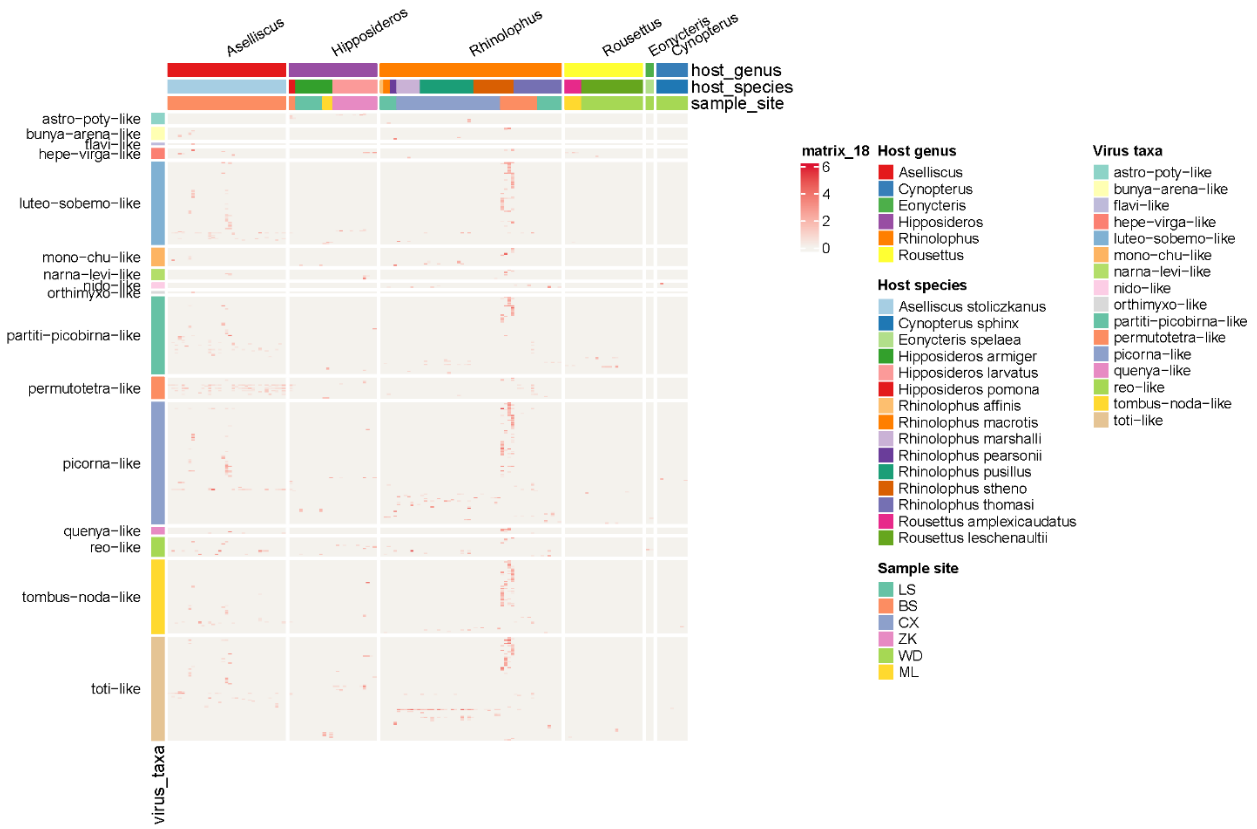

Supplementary Fig. 5 | Characterization of the total RNA virome of bats. The heatmap displays the distribution and abundance of total RNA viruses in individual bats. Each column represents an individual bat, while each row represents a virus species. The abundance of viruses in each individual is represented as a logarithm of the number of mapped reads per million total reads (RPM). Sampling site, host taxonomy (species and genus) and virus taxonomy are shown as coloured strips at top and left, respectively.

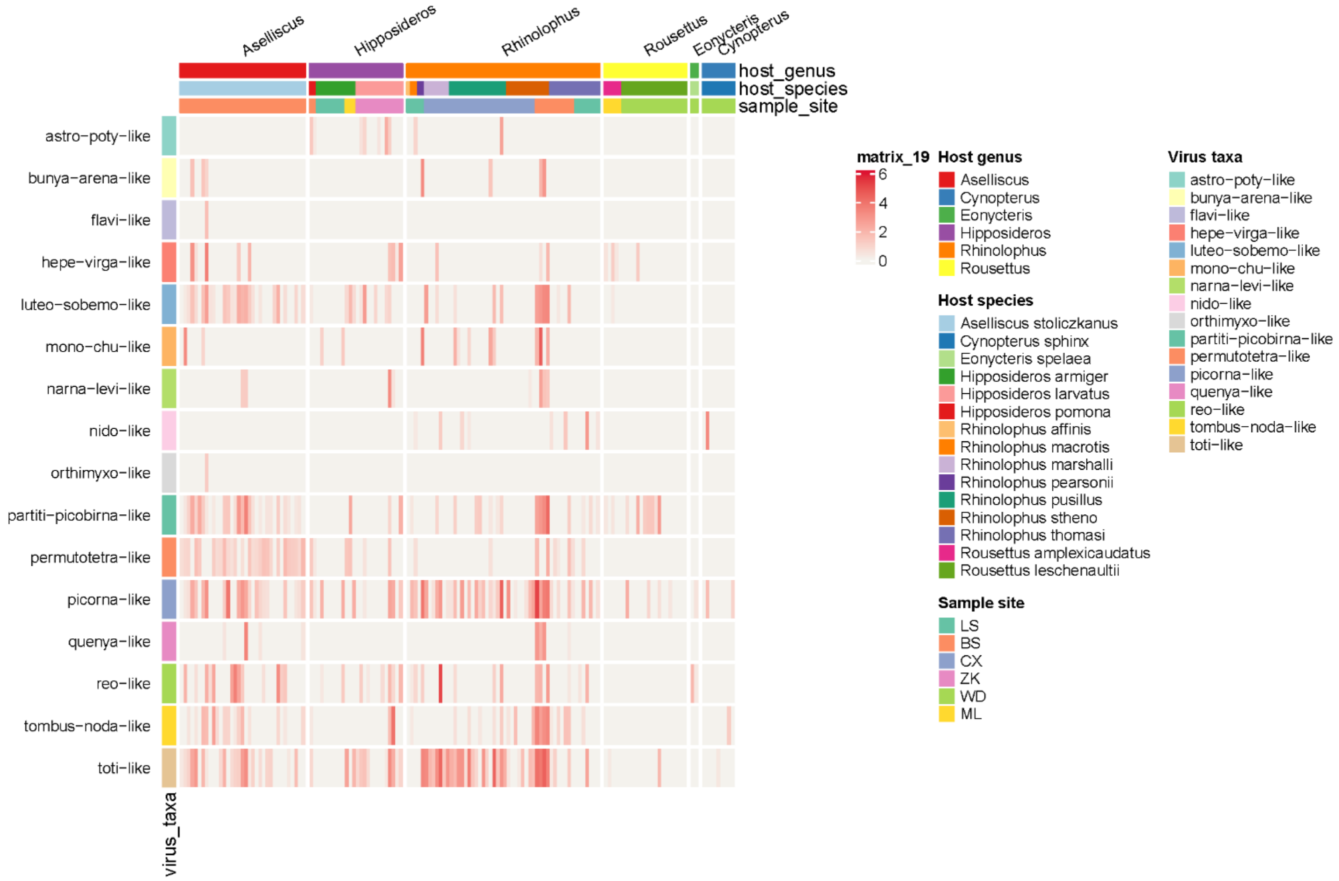

Supplementary Fig. 6 | Characterization of the total RNA virome of bats. In this figure, viruses are collapsed by order, i.e., each row represents a viral order.

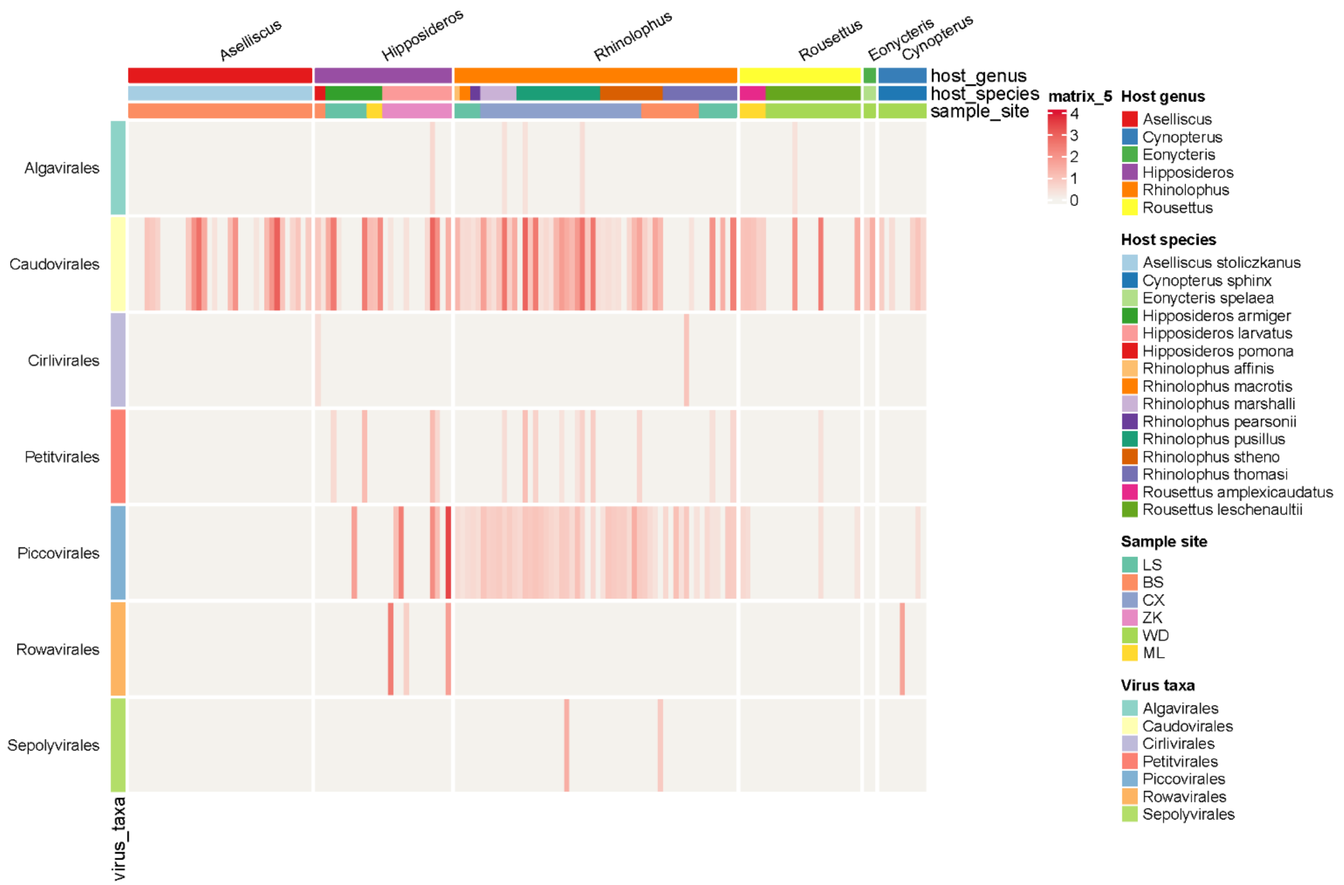

Supplementary Fig. 7 | Characterization of the total DNA virome of bats. In this figure, viruses are collapsed by order, i.e., each row represents a viral order.

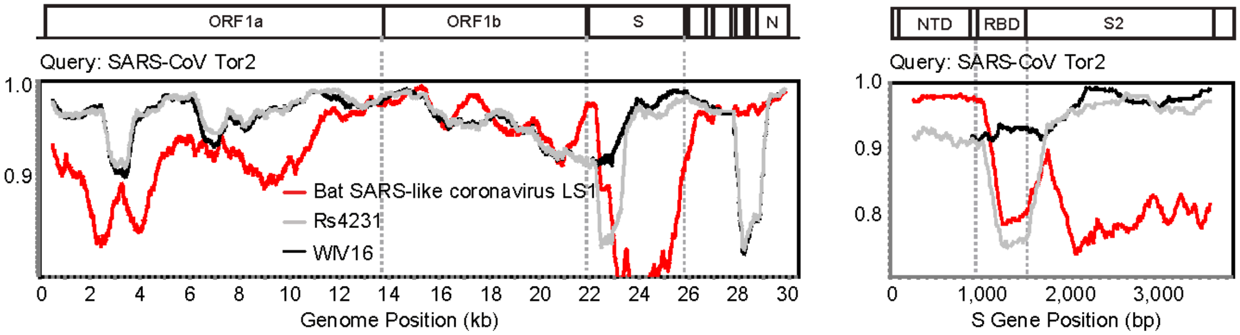

Supplementary Fig. 8 | Recombination analysis of SARS-related coronaviruses at the whole genome and spike protein scales.

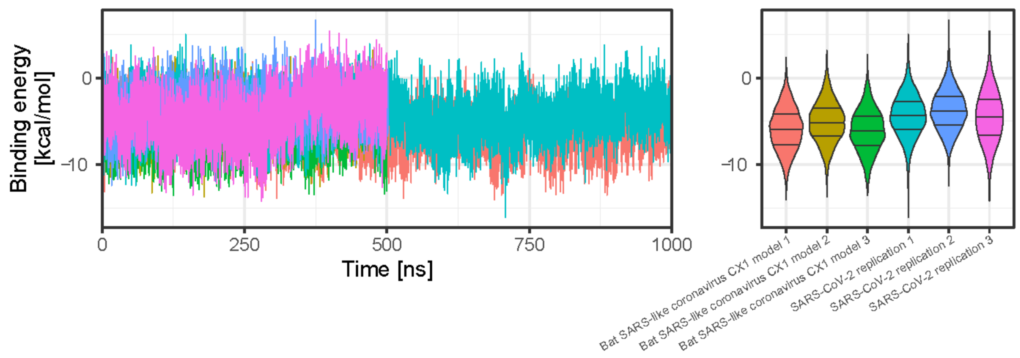

Supplementary Fig. 9 | The predicted binding energy of the RBD-hACE2 complex in the duration of MD simulations. Two 1000-ns-long main simulations and four 500-ns-long replications were performed. These results showed that Bat SARS-like coronavirus CX1 RBD can consistently bind to hACE2, although the binding energy is slightly lower than human SARS-CoV-2, suggesting higher affinity.

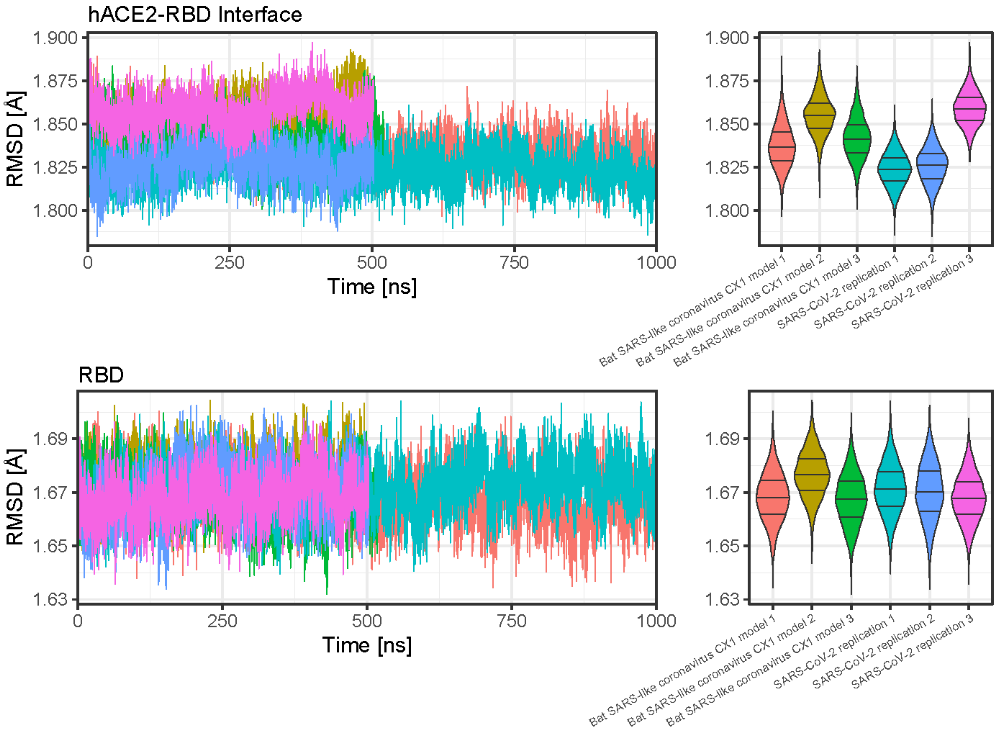

Supplementary Fig. 10 | The deviation in protein backbone position during the MD simulations. RMSD is the abbreviation of root-mean-square deviation of atomic positions. We used backbone (C, N and O atoms in the main chain) RMSD to reflect the stability of RBD-hACE2 binding. Bat SARS-like coronavirus CX1 displayed similar binding stability with human SARS-CoV-2, regarding the RBD and RBD-hACE2 interface.

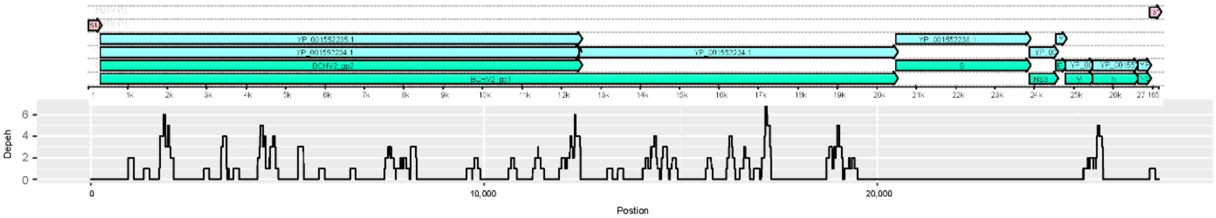

Supplementary Fig. 11 | The genome coverage of HKU2-like LS1 from the library of S18LSBatR79. The reference genome was Rhinolophus bat coronavirus HKU2 (NC_009988.1).

Supplementary Tables

Supplementary Table 1. Details of the 149 bats samples and reads counts of corresponding meta-transcriptomic sequencing libraries.

lib_id	species	genus	family	year	Sample site	clean_reads	norRNA_reads
S17BSBatR15	*Aselliscus stoliczkanus*	*Aselliscus*	Hipposideridae	2017	BS	68880512	44506938
S17BSBatR16	*Aselliscus stoliczkanus*	*Aselliscus*	Hipposideridae	2017	BS	75999644	34192819
S17BSBatR48	*Aselliscus stoliczkanus*	*Aselliscus*	Hipposideridae	2017	BS	71871898	33072042
S17BSBatR06	*Aselliscus stoliczkanus*	*Aselliscus*	Hipposideridae	2017	BS	74929166	27267536
S17BSBatR07	*Aselliscus stoliczkanus*	*Aselliscus*	Hipposideridae	2017	BS	96947200	34805448
S17BSBatR08	*Aselliscus stoliczkanus*	*Aselliscus*	Hipposideridae	2017	BS	91702380	51779185
S15BSBatR02	*Aselliscus stoliczkanus*	*Aselliscus*	Hipposideridae	2015	BS	70860150	54619603
S15BSBatR03	*Aselliscus stoliczkanus*	*Aselliscus*	Hipposideridae	2015	BS	72453364	50879141
S17BSBatR41	*Aselliscus stoliczkanus*	*Aselliscus*	Hipposideridae	2017	BS	80656886	59747842
S17BSBatR42	*Aselliscus stoliczkanus*	*Aselliscus*	Hipposideridae	2017	BS	66525248	26985400
S17BSBatR43	*Aselliscus stoliczkanus*	*Aselliscus*	Hipposideridae	2017	BS	93421078	70656412
S17BSBatR44	*Aselliscus stoliczkanus*	*Aselliscus*	Hipposideridae	2017	BS	69262522	28294702
S17BSBatR45	*Aselliscus stoliczkanus*	*Aselliscus*	Hipposideridae	2017	BS	90277278	18371471
S17BSBatR46	*Aselliscus stoliczkanus*	*Aselliscus*	Hipposideridae	2017	BS	65986080	19538297
S17BSBatR47	*Aselliscus stoliczkanus*	*Aselliscus*	Hipposideridae	2017	BS	84954490	33231584
S17BSBatR49	*Aselliscus stoliczkanus*	*Aselliscus*	Hipposideridae	2017	BS	93813908	44028166
S15BSBatR07	*Aselliscus stoliczkanus*	*Aselliscus*	Hipposideridae	2015	BS	75906002	41793266
S15BSBatR08	*Aselliscus stoliczkanus*	*Aselliscus*	Hipposideridae	2015	BS	85418074	62171363
S15BSBatR09	*Aselliscus stoliczkanus*	*Aselliscus*	Hipposideridae	2015	BS	81566458	58015712
S17BSBatR17	*Aselliscus stoliczkanus*	*Aselliscus*	Hipposideridae	2017	BS	82798246	28358352
S17BSBatR18	*Aselliscus stoliczkanus*	*Aselliscus*	Hipposideridae	2017	BS	68499666	41435388
S17BSBatR19	*Aselliscus stoliczkanus*	*Aselliscus*	Hipposideridae	2017	BS	86115436	46209043
S17BSBatR20	*Aselliscus stoliczkanus*	*Aselliscus*	Hipposideridae	2017	BS	93076172	48944765
S17BSBatR04	*Aselliscus stoliczkanus*	*Aselliscus*	Hipposideridae	2017	BS	75987416	50810480
S17BSBatR31	*Aselliscus stoliczkanus*	*Aselliscus*	Hipposideridae	2017	BS	96054938	47400247
S17BSBatR33	*Aselliscus stoliczkanus*	*Aselliscus*	Hipposideridae	2017	BS	71969046	20304203
S17BSBatR34	*Aselliscus stoliczkanus*	*Aselliscus*	Hipposideridae	2017	BS	67853898	50560539
S17BSBatR35	*Aselliscus stoliczkanus*	*Aselliscus*	Hipposideridae	2017	BS	78930206	60628177
S17BSBatR36	*Aselliscus stoliczkanus*	*Aselliscus*	Hipposideridae	2017	BS	71511338	54127554
S17BSBatR37	*Aselliscus stoliczkanus*	*Aselliscus*	Hipposideridae	2017	BS	81524164	42966608
S17BSBatR38	*Aselliscus stoliczkanus*	*Aselliscus*	Hipposideridae	2017	BS	88633424	51078488
S17BSBatR39	*Aselliscus stoliczkanus*	*Aselliscus*	Hipposideridae	2017	BS	84276296	53346626
S17BSBatR50	*Aselliscus stoliczkanus*	*Aselliscus*	Hipposideridae	2017	BS	73817862	35687375
S17BSBatR09	*Aselliscus stoliczkanus*	*Aselliscus*	Hipposideridae	2017	BS	67861376	36100117
S17BSBatR10	*Aselliscus stoliczkanus*	*Aselliscus*	Hipposideridae	2017	BS	70281582	41017175
S17WDBatR05	*Cynopterus sphinx*	*Cynopterus*	Pteropodidae	2017	WD	73614966	56763613
S16WDBatR58	*Cynopterus sphinx*	*Cynopterus*	Pteropodidae	2016	WD	68808808	38117763

S17WDBatR04	*Cynopterus sphinx*	*Cynopterus*	Pteropodidae	2017	WD	67191458	42223044
S17WDBatR08	*Cynopterus sphinx*	*Cynopterus*	Pteropodidae	2017	WD	67293178	26546783
S17WDBatR03	*Cynopterus sphinx*	*Cynopterus*	Pteropodidae	2017	WD	67958666	41309320
S18WDBatR01	*Cynopterus sphinx*	*Cynopterus*	Pteropodidae	2018	WD	83891592	44088306
S17WDBatR06	*Cynopterus sphinx*	*Cynopterus*	Pteropodidae	2017	WD	70939608	35259402
S17WDBatR09	*Cynopterus sphinx*	*Cynopterus*	Pteropodidae	2017	WD	68646994	32378944
S16WDBatR60	*Cynopterus sphinx*	*Cynopterus*	Pteropodidae	2016	WD	67517154	38318585
S16WDBatR61	*Eonycteris spelaea*	*Eonycteris*	Pteropodidae	2016	WD	75078882	32106839
S18WDBatR02	*Eonycteris spelaea*	*Eonycteris*	Pteropodidae	2018	WD	74989214	30481633
S17BSBatR05	*Hipposideros pomona*	*Hipposideros*	Hipposideridae	2017	BS	71509188	15704666
S17BSBatR03	*Hipposideros pomona*	*Hipposideros*	Hipposideridae	2017	BS	68499306	34969863
S18LSBatR57	*Hipposideros armiger*	*Hipposideros*	Hipposideridae	2018	LS	65950060	47658214
S18LSBatR58	*Hipposideros armiger*	*Hipposideros*	Hipposideridae	2018	LS	68459604	56279452
S18LSBatR53	*Hipposideros armiger*	*Hipposideros*	Hipposideridae	2018	LS	67177820	48054491
S18LSBatR54	*Hipposideros armiger*	*Hipposideros*	Hipposideridae	2018	LS	68770812	38631306
S18LSBatR55	*Hipposideros armiger*	*Hipposideros*	Hipposideridae	2018	LS	71462950	34852173
S18LSBatR56	*Hipposideros armiger*	*Hipposideros*	Hipposideridae	2018	LS	67251454	51315508
S18LSBatR60	*Hipposideros armiger*	*Hipposideros*	Hipposideridae	2018	LS	71576454	53220809
S18LSBatR59	*Hipposideros armiger*	*Hipposideros*	Hipposideridae	2018	LS	69240450	55171410
S19MLBatR10	*Hipposideros armiger*	*Hipposideros*	Hipposideridae	2019	ML	76686836	49786405
S19MLBatR09	*Hipposideros armiger*	*Hipposideros*	Hipposideridae	2019	ML	78112840	29749427
S19MLBatR11	*Hipposideros armiger*	*Hipposideros*	Hipposideridae	2019	ML	66418404	31525709
S15ZKBatR23	*Hipposideros larvatus*	*Hipposideros*	Hipposideridae	2015	ZK	67487566	54870198
S15ZKBatR21	*Hipposideros larvatus*	*Hipposideros*	Hipposideridae	2015	ZK	83156356	59230411
S15ZKBatR22	*Hipposideros larvatus*	*Hipposideros*	Hipposideridae	2015	ZK	70537570	61027468
S15ZKBatR24	*Hipposideros larvatus*	*Hipposideros*	Hipposideridae	2015	ZK	77064058	59641649
S15ZKBatR25	*Hipposideros larvatus*	*Hipposideros*	Hipposideridae	2015	ZK	70955122	63091112
S15ZKBatR26	*Hipposideros larvatus*	*Hipposideros*	Hipposideridae	2015	ZK	71540842	60869301
S15ZKBatR27	*Hipposideros larvatus*	*Hipposideros*	Hipposideridae	2015	ZK	75954134	63685002
S15ZKBatR28	*Hipposideros larvatus*	*Hipposideros*	Hipposideridae	2015	ZK	77997366	61943057
S15ZKBatR29	*Hipposideros larvatus*	*Hipposideros*	Hipposideridae	2015	ZK	99891834	72052070
S15ZKBatR30	*Hipposideros larvatus*	*Hipposideros*	Hipposideridae	2015	ZK	74775210	35351897
S15ZKBatR11	*Hipposideros larvatus*	*Hipposideros*	Hipposideridae	2015	ZK	72762514	12105852
S15ZKBatR12	*Hipposideros larvatus*	*Hipposideros*	Hipposideridae	2015	ZK	66829092	58012754
S15ZKBatR13	*Hipposideros larvatus*	*Hipposideros*	Hipposideridae	2015	ZK	54362176	34078817
S18LSBatR81	*Rhinolophus affinis*	*Rhinolophus*	Rhinolophidae	2018	LS	49475970	20705771
S18LSBatR74	*Rhinolophus macrotis*	*Rhinolophus*	Rhinolophidae	2018	LS	60060528	24057082
S18LSBatR73	*Rhinolophus macrotis*	*Rhinolophus*	Rhinolophidae	2018	LS	55214320	15962511
S18LSBatR76	*Rhinolophus pearsonii*	*Rhinolophus*	Rhinolophidae	2018	LS	58997270	12254475
S18LSBatR78	*Rhinolophus pearsonii*	*Rhinolophus*	Rhinolophidae	2018	LS	49224150	7420822
S18CXBatR07	*Rhinolophus marshalli*	*Rhinolophus*	Rhinolophidae	2018	CX	69621878	11273318
S18CXBatR19	*Rhinolophus marshalli*	*Rhinolophus*	Rhinolophidae	2018	CX	1.14E+08	96844036

S18CXBatR04	*Rhinolophus marshalli*	*Rhinolophus*	Rhinolophidae	2018	CX	71911444	59771830
S18CXBatR05	*Rhinolophus marshalli*	*Rhinolophus*	Rhinolophidae	2018	CX	68241790	27343221
S18CXBatR24	*Rhinolophus marshalli*	*Rhinolophus*	Rhinolophidae	2018	CX	1.06E+08	10758449
S18CXBatR26	*Rhinolophus marshalli*	*Rhinolophus*	Rhinolophidae	2018	CX	1.19E+08	1.02E+08
S18CXBatR27	*Rhinolophus marshalli*	*Rhinolophus*	Rhinolophidae	2018	CX	1.12E+08	18265891
S18CXBatR10	*Rhinolophus pusillus*	*Rhinolophus*	Rhinolophidae	2018	CX	63379146	28506295
S18CXBatR21	*Rhinolophus pusillus*	*Rhinolophus*	Rhinolophidae	2018	CX	1.03E+08	10741787
S18CXBatR16	*Rhinolophus pusillus*	*Rhinolophus*	Rhinolophidae	2018	CX	88419254	54559586
S18CXBatR29	*Rhinolophus pusillus*	*Rhinolophus*	Rhinolophidae	2018	CX	1.09E+08	18103956
S18CXBatR20	*Rhinolophus pusillus*	*Rhinolophus*	Rhinolophidae	2018	CX	1.67E+08	1.32E+08
S18CXBatR09	*Rhinolophus pusillus*	*Rhinolophus*	Rhinolophidae	2018	CX	99403952	47915468
S18CXBatR02	*Rhinolophus pusillus*	*Rhinolophus*	Rhinolophidae	2018	CX	67996538	48698414
S18CXBatR15	*Rhinolophus pusillus*	*Rhinolophus*	Rhinolophidae	2018	CX	1.31E+08	11192628
S18CXBatR22	*Rhinolophus pusillus*	*Rhinolophus*	Rhinolophidae	2018	CX	1.08E+08	42138869
S18CXBatR14-2	*Rhinolophus pusillus*	*Rhinolophus*	Rhinolophidae	2018	CX	1.07E+08	37667832
S18CXBatR12	*Rhinolophus pusillus*	*Rhinolophus*	Rhinolophidae	2018	CX	1.05E+08	40302452
S18CXBatR13	*Rhinolophus pusillus*	*Rhinolophus*	Rhinolophidae	2018	CX	1.13E+08	10148950
S18CXBatR23	*Rhinolophus pusillus*	*Rhinolophus*	Rhinolophidae	2018	CX	1.14E+08	7305986
S18CXBatR25	*Rhinolophus pusillus*	*Rhinolophus*	Rhinolophidae	2018	CX	1.14E+08	81392657
S18CXBatR30	*Rhinolophus pusillus*	*Rhinolophus*	Rhinolophidae	2018	CX	1.16E+08	6529464
S18CXBatR31	*Rhinolophus pusillus*	*Rhinolophus*	Rhinolophidae	2018	CX	1E+08	45270117
S18CXBatR08	*Rhinolophus stheno*	*Rhinolophus*	Rhinolophidae	2018	CX	75384154	16447965
S18CXBatR18	*Rhinolophus stheno*	*Rhinolophus*	Rhinolophidae	2018	CX	1.17E+08	88625344
S18CXBatR01	*Rhinolophus stheno*	*Rhinolophus*	Rhinolophidae	2018	CX	1.16E+08	93201201
S18CXBatR03	*Rhinolophus stheno*	*Rhinolophus*	Rhinolophidae	2018	CX	67604788	54282166
S18CXBatR06	*Rhinolophus stheno*	*Rhinolophus*	Rhinolophidae	2018	CX	80760756	60095676
S18CXBatR28	*Rhinolophus stheno*	*Rhinolophus*	Rhinolophidae	2018	CX	1.13E+08	71799955
S18CXBatR17-2	*Rhinolophus stheno*	*Rhinolophus*	Rhinolophidae	2018	CX	1.08E+08	38124264
S18CXBatR11	*Rhinolophus stheno*	*Rhinolophus*	Rhinolophidae	2018	CX	1.26E+08	19196122
S15BSBatR06	*Rhinolophus stheno*	*Rhinolophus*	Rhinolophidae	2015	BS	65314642	25746124
S15BSBatR05	*Rhinolophus stheno*	*Rhinolophus*	Rhinolophidae	2015	BS	65305160	18771296
S15BSBatR01	*Rhinolophus stheno*	*Rhinolophus*	Rhinolophidae	2015	BS	71119598	15944072
S15BSBatR04	*Rhinolophus stheno*	*Rhinolophus*	Rhinolophidae	2015	BS	64889926	24127736
S17BSBatR11	*Rhinolophus thomasi*	*Rhinolophus*	Rhinolophidae	2017	BS	65440252	38926950
S17BSBatR13	*Rhinolophus thomasi*	*Rhinolophus*	Rhinolophidae	2017	BS	67377824	46518061
S17BSBatR14	*Rhinolophus thomasi*	*Rhinolophus*	Rhinolophidae	2017	BS	65348384	33970555
S17BSBatR01	*Rhinolophus thomasi*	*Rhinolophus*	Rhinolophidae	2017	BS	58241216	39602737
S17BSBatR02	*Rhinolophus thomasi*	*Rhinolophus*	Rhinolophidae	2017	BS	66765760	43411641
S17BSBatR12	*Rhinolophus thomasi*	*Rhinolophus*	Rhinolophidae	2017	BS	74803632	28579849
S17BSBatR40	*Rhinolophus thomasi*	*Rhinolophus*	Rhinolophidae	2017	BS	73270944	44245627
S18LSBatR77	*Rhinolophus thomasi*	*Rhinolophus*	Rhinolophidae	2018	LS	56022704	30102862
S18LSBatR80	*Rhinolophus thomasi*	*Rhinolophus*	Rhinolophidae	2018	LS	44192582	28709370

S18LSBatR82	*Rhinolophus thomasi*	*Rhinolophus*	Rhinolophidae	2018	LS	47203674	23348871
S18LSBatR79	*Rhinolophus thomasi*	*Rhinolophus*	Rhinolophidae	2018	LS	52330550	23436743
S18LSBatR75	*Rhinolophus thomasi*	*Rhinolophus*	Rhinolophidae	2018	LS	54469990	17648763
S18LSBatR62	*Rhinolophus thomasi*	*Rhinolophus*	Rhinolophidae	2018	LS	73711364	58724419
S18LSBatR61	*Rhinolophus thomasi*	*Rhinolophus*	Rhinolophidae	2018	LS	77023514	26512424
S19MLBatR14	*Rousettus amplexicaudatus*	*Rousettus*	Pteropodidae	2019	ML	78082162	17863826
S19MLBatR12	*Rousettus amplexicaudatus*	*Rousettus*	Pteropodidae	2019	ML	98127700	47140492
S19MLBatR13	*Rousettus amplexicaudatus*	*Rousettus*	Pteropodidae	2019	ML	76305938	16738464
S19MLBatR15	*Rousettus amplexicaudatus*	*Rousettus*	Pteropodidae	2019	ML	75387638	31881753
S19MLBatR16	*Rousettus amplexicaudatus*	*Rousettus*	Pteropodidae	2019	ML	65162828	45542147
S17WDBatR01	*Rousettus leschenaultii*	*Rousettus*	Pteropodidae	2017	WD	72177110	51281139
S17WDBatR10	*Rousettus leschenaultii*	*Rousettus*	Pteropodidae	2017	WD	75110590	48582603
S16WDBatR55	*Rousettus leschenaultii*	*Rousettus*	Pteropodidae	2016	WD	78537748	54648732
S16WDBatR59	*Rousettus leschenaultii*	*Rousettus*	Pteropodidae	2016	WD	75013354	42808530
S17WDBatR02	*Rousettus leschenaultii*	*Rousettus*	Pteropodidae	2017	WD	66785654	45208328
S18WDBatR03	*Rousettus leschenaultii*	*Rousettus*	Pteropodidae	2018	WD	68894874	52781177
S18WDBatR04	*Rousettus leschenaultii*	*Rousettus*	Pteropodidae	2018	WD	75336460	52153071
S18WDBatR05	*Rousettus leschenaultii*	*Rousettus*	Pteropodidae	2018	WD	67836198	38900686
S18WDBatR06	*Rousettus leschenaultii*	*Rousettus*	Pteropodidae	2018	WD	98176992	63451329
S18WDBatR07	*Rousettus leschenaultii*	*Rousettus*	Pteropodidae	2018	WD	70884072	52972290
S18WDBatR08	*Rousettus leschenaultii*	*Rousettus*	Pteropodidae	2018	WD	64049926	8513037
S18WDBatR09	*Rousettus leschenaultii*	*Rousettus*	Pteropodidae	2018	WD	92628194	68038890
S18WDBatR10	*Rousettus leschenaultii*	*Rousettus*	Pteropodidae	2018	WD	66267308	49915815
S17WDBatR07	*Rousettus leschenaultii*	*Rousettus*	Pteropodidae	2017	WD	71526006	55738841
S16WDBatR56	*Rousettus leschenaultii*	*Rousettus*	Pteropodidae	2016	WD	68397658	53572725
S16WDBatR57	*Rousettus leschenaultii*	*Rousettus*	Pteropodidae	2016	WD	75851196	32004029
S16WDBatR62	*Rousettus leschenaultii*	*Rousettus*	Pteropodidae	2016	WD	67326708	57273260
S16WDBatR63	*Rousettus leschenaultii*	*Rousettus*	Pteropodidae	2016	WD	65393626	37310541

Supplementary Table 2. The 55 mammal-associated viruses identified in this study.

virus_name_new	family	num_host_species	num_host_genus	virus_of_concern	Novel virus species	Blastx hit	Acesssion number	E value	Identity
Bat astrovirus ZK1	*Astroviridae*	1	1	No	Yes	Bat astrovirus	QVW10123.1	8.51E-208	55.1
Bat astrovirus BS1	*Astroviridae*	1	1	No	Yes	Bat astrovirus Ha/Guangxi/LS11/2007	ACN88713.1	1.93E-142	80.9
Bat astrovirus LS1	*Astroviridae*	1	1	No	Yes	Bat astrovirus Ha/Guangxi/LS11/2007	ACN88713.1	2.45E-255	78.4
Bat anellovirus ZK1	*Anelloviridae*	1	1	No	Yes	Torque teno felis virus	QZE11920.1	5.00E-26	30.94
Bat astrovirus ZK2	*Astroviridae*	1	1	No	Yes	Bat astrovirus	QOR29562.1	0	61.81
Bat astrovirus BS2	*Astroviridae*	1	1	No	Yes	Bat astrovirus Ha/Guangxi/LS11/2007	ACN88713.1	0	82.62
Bat astrovirus BS3	*Astroviridae*	1	1	No	Yes	Bat astrovirus Ha/Guangxi/LS11/2007	ACN88713.1	0	81.3
Bat astrovirus CX1	*Astroviridae*	1	1	No	Yes	Bat astrovirus Ha/Guangxi/LS11/2007	ACN88713.1	0	77.13
Bat astrovirus CX2	*Astroviridae*	1	1	No	Yes	Bat astrovirus Ha/Guangxi/LS11/2007	ACN88713.1	0	79.55
Bat bocaparvovirus ZK1	*Parvoviridae*	2	1	No	Yes	Rhinolophus sinicus bocaparvovirus	AOX47676.1	0	68.54
Bat circovirus CX1	*Cicoviridae*	1	1	No	Yes	Bat associated circovirus 9	YP_009508628.1	0	78.46
Bat coronavirus WD2	*Coronaviridae*	1	1	No	No	Coronavirus BtRt-BetaCoV/GX2018	QDF43839.1	0	99.15
Bat HKU10-like coronavirus BS1	*Coronaviridae*	1	1	No	No	Hipposideros bat coronavirus HKU10	AFU92121.1	0	98.99
Bat mastadenovirus WD1	*Adenoviridae*	1	1	No	Yes	Bat mastadenovirus B	QDA77076.1	0	55.87
Bat mastadenovirus ZK1	*Adenoviridae*	1	1	No	Yes	Equine adenovirus 1	ANG08548.1	0	65.43
Bat pararubulavirus WD1	*Paramyxoviridae*	1	1	No	Yes	Bat paramyxovirus	AIF74183.1	0	83.23
Bat parvovirus CX1	*Parvoviridae*	1	1	No	Yes	Bat parvovirus	QRV11697.1	0	49.52
Bat parvovirus BS1	*Parvoviridae*	1	1	No	Yes	Bat parvovirus	QRV11697.1	0	51.68
Bat parvovirus LS1	*Parvoviridae*	1	1	No	Yes	Bat parvovirus	AIF74198.1	0	78.83
Bat picornavirus CX7	*Picornaviridae*	1	1	No	Yes	Bat picornavirus 3	YP_004782204.1	0	55.95
Bat picornavirus CX3	*Picornaviridae*	1	1	No	Yes	Teschovirus sp.	ART66861.1	0	64.93
Bat picornavirus WD3	*Picornaviridae*	1	1	No	Yes	Bat picornavirus	AIF74257.1	0	58.21
Bat picornavirus CX1	*Picornaviridae*	1	1	No	Yes	Bat picornavirus	AIF74257.1	0	54.24
Bat picornavirus CX4	*Picornaviridae*	2	1	No	Yes	Canine picornavirus	AMX81410.1	0	67.72
Bat picornavirus CX2	*Picornaviridae*	2	1	No	Yes	Bat picornavirus CUS8	QTE76071.1	0	54.82
Bat picornavirus ZK1	*Picornaviridae*	1	1	No	Yes	Bat picornavirus LMA6	QTE76069.1	0	54.48
Bat picornavirus CX5	*Picornaviridae*	2	1	No	Yes	Bat picornavirus CUS8	QTE76071.1	0	51.16
Bat polyomavirus CX1	*Polyomaviridae*	2	2	No	No	Bat polyomavirus	AIF74274.1	0	99.64

Bat RVJ-like rotavirus ZK1	*Reoviridae*	1	1	No	Yes	Rotavirus J	YP_010086029.1	0	74.48
Bat RVJ-like rotavirus CX2	*Reoviridae*	1	1	No	Yes	Rotavirus J	YP_010086029.1	0	67.69
Bat RVJ-like rotavirus LS1	*Reoviridae*	1	1	No	Yes	Rotavirus J	YP_010086029.1	0	68.97
Bat sapovirus WD1	*Caliciviridae*	1	1	No	Yes	Bat sapovirus	YP_009345598.1	0	40.63
Bat sapovirus WD2	*Caliciviridae*	1	1	No	Yes	Bat sapovirus	YP_009345598.1	0	59.09
Bat sapovirus WD3	*Caliciviridae*	2	1	No	Yes	Bat sapovirus	YP_009345598.1	0	45.22
Bat SARS-like coronavirus LS1	*Coronaviridae*	2	1	Yes	No	BtRs-BetaCoV/GX2013	AIA62319.1	0	99.47
Bat SARS-like coronavirus CX1	*Coronaviridae*	2	1	Yes	No	Bat SARS-like coronavirus RsSHC014	AGZ48805.1	0	99.34
Bat orthoreovirus BS1	*Reoviridae*	4	3	Yes	No	Mammalian orthoreovirus	AXM44651.1	0	99.43
Bat tupavirus BS1	*Rhabdoviridae*	1	1	No	Yes	Wufeng Rhinolophus pearsonii tupavirus 1	UBB42393.1	0	75.6
Bat tupavirus BS2	*Rhabdoviridae*	1	1	No	Yes	Wufeng Rhinolophus pearsonii tupavirus 1	UBB42393.1	0	72.5
Bat tupavirus CX1	*Rhabdoviridae*	1	1	No	Yes	Klamath virus	YP_009362266.1	4.24E-286	63.6
Bat tupavirus CX2	*Rhabdoviridae*	1	1	No	Yes	Klamath virus	YP_009362266.1	0	62
Bat tupavirus CX3	*Rhabdoviridae*	1	1	No	Yes	Wufeng Rhinolophus pearsonii tupavirus 1	UBB42393.1	0	76.3
Bat picornavirus WD1	*Picornaviridae*	1	1	No	Yes	Rhinolophus sinicus picornavirus	AWK02666.1	1.13E-198	57.9
Bat picornavirus BS1	*Picornaviridae*	1	1	No	Yes	Rhinolophus picornavirus	AWK02675.1	0	71.7
Bat picornavirus BS2	*Picornaviridae*	1	1	No	No	Rhinolophus sinicus picornavirus	AWK02666.1	0	92.9
Bat picornavirus CX8	*Picornaviridae*	1	1	No	Yes	Bat picornavirus CUS8	QTE76071.1	0	53.8
Bat picornavirus CX9	*Picornaviridae*	1	1	No	Yes	Bat picornavirus	AIF74256.1	1.63E-279	72.2
Bat picornavirus LS1	*Picornaviridae*	1	1	No	Yes	Rhinolophus sinicus picornavirus	AWK02666.1	0	67.1
Bat picornavirus WD2	*Picornaviridae*	1	1	No	Yes	Crohivirus B	YP_009345900.1	1.32E-151	59.8
Bat HKU2-like alphacoronavirus LS1	*Coronaviridae*	1	1	Yes	No	Rhinolophus bat coronavirus HKU2	ABB77049.1	7.00E-140	99.51
Bat rotavirus A type CX1	*Reoviridae*	5	2	Yes	No	Simian rotavirus A strain TUCH	B3F2X5.1	0	98.81
Bat rotavirus A type WD1	*Reoviridae*	1	1	No	No	Rotavirus A	ARB49202.1	0	90.91
Bat rotavirus A type WD2	*Reoviridae*	1	1	No	No	Rotavirus A	ARB49202.1	0	96.88
Bat HKU9-like betacoronavirus WD1	*Coronaviridae*	1	1	No	No	Bat coronavirus HKU9-5-2	ADM33565.1	0	99.34
Bat picornavirus CX6	*Picornaviridae*	1	1	No	No	Bat picornavirus	AIF74257.1	0	98.46

Supplementary Table 3. Viral genome coverage and read depth statistics. "reference_length" column refers to the genome size of the most closely related reference genome, and "coverage_ref" is percentage coverage relative to that reference genome.

lib_id	virus_name	RPM	numreads	covbases	meandepth	reference_length	coverage_ref
S15ZKBatR30	Bat anellovirus ZK1	4.44	163	1064	21.48	2367	45.00%
S17BSBatR05	Bat astrovirus BS1	4.97	132	1340	11.10	6813	19.70%
S17BSBatR03	Bat astrovirus BS1	1.09	51	961	4.37	6813	14.10%
S17BSBatR05	Bat astrovirus BS2	5.79	105	1114	12.67	6813	16.40%
S17BSBatR05	Bat astrovirus BS3	11.91	196	1998	13.92	6813	29.30%
S18CXBatR30	Bat astrovirus CX1	251.93	1668	3900	62.45	6813	57.20%
S18CXBatR30	Bat astrovirus CX2	122.67	813	2446	48.51	6813	35.90%
S18LSBatR73	Bat astrovirus LS1	8.14	130	1279	14.91	6813	18.80%
S15ZKBatR30	Bat astrovirus ZK1	10.18	361	2703	19.15	6813	39.70%
S15ZKBatR29	Bat astrovirus ZK2	174.96	12639	6529	276.22	6813	95.80%
S15ZKBatR22	Bat astrovirus ZK2	7.72	473	6400	10.12	6813	93.90%
S15ZKBatR21	Bat astrovirus ZK2	2.33	147	5398	3.10	6813	79.20%
S15ZKBatR30	Bat astrovirus ZK2	4.89	176	4113	3.81	6813	60.40%
S15ZKBatR27	Bat astrovirus ZK2	1.30	88	3237	1.92	6813	47.50%
S15ZKBatR24	Bat bocaparvovirus ZK1	307.22	11621	2322	668.95	4880	47.60%
S18LSBatR56	Bat bocaparvovirus ZK1	61.48	966	1976	49.91	4880	40.50%
S15ZKBatR22	Bat bocaparvovirus ZK1	11.95	285	1960	16.17	4880	40.20%
S18LSBatR57	Bat bocaparvovirus ZK1	1.09	22	1007	1.28	4880	20.60%
S18CXBatR22	Bat circovirus CX1	13.17	593	1816	46.63	1816	100.00%
S17BSBatR02	Bat HKU10-like coronavirus BS1	51.16	2859	6364	60.35	28494	22.30%
S17BSBatR11	Bat HKU10-like coronavirus BS1	5.09	246	5965	5.26	28494	20.90%
S18LSBatR79	Bat HKU10-like coronavirus BS1	10.24	310	5738	6.74	28494	20.10%
S17BSBatR12	Bat HKU10-like coronavirus BS1	2.27	74	4171	1.58	28494	14.60%
S18CXBatR09	Bat HKU10-like coronavirus BS1	1.52	167	4160	3.49	28494	14.60%
S18CXBatR13	Bat HKU2-like coronavirus LS1	1.58	18	855	0.84	27165	3.10%
S17WDBatR02	Bat HKU9-like coronavirus WD1	12.08	590	19849	2.86	29114	68.20%
S17WDBatR07	Bat HKU9-like coronavirus WD1	3.64	210	12728	1.02	29114	43.70%
S18WDBatR05	Bat HKU9-like coronavirus WD1	1.03	40	2032	0.19	29114	7.00%
S16WDBatR58	Bat coronavirus WD2	2156.74	86662	30208	402.15	29114	100.00%
S17WDBatR03	Bat mastadenovirus WD1	5.79	240	2681	12.63	29812	9.00%
S15ZKBatR21	Bat mastadenovirus ZK1	219.60	13097	13159	139.74	29812	44.10%
S15ZKBatR13	Bat mastadenovirus ZK1	35.74	1273	12620	12.69	29812	42.30%
S15ZKBatR25	Bat mastadenovirus ZK1	3.19	204	8841	2.11	29812	29.70%
S17BSBatR47	Bat orthoreovirus BS1	2318.13	78857	23245	510.76	23200	100.00%
S17BSBatR04	Bat orthoreovirus BS1	17.71	924	21513	5.75	23200	92.70%

S17BSBatR42	Bat orthoreovirus BS1	946.40	32078	20191	225.21	23200	87.00%
S18LSBatR59	Bat orthoreovirus BS1	44.62	3183	15785	21.64	23200	68.00%
S18LSBatR58	Bat orthoreovirus BS1	2.35	189	9478	1.49	23200	40.90%
S18CXBatR01	Bat orthoreovirus BS1	1.31	146	9042	1.19	23200	39.00%
S18CXBatR11	Bat orthoreovirus BS1	1.41	38	1610	0.51	23200	6.90%
S15ZKBatR11	Bat orthoreovirus BS1	2.15	32	1190	0.39	23200	5.10%
S16WDBatR58	Bat pararubulavirus WD1	3.49	135	1315	14.54	15888	8.30%
S17BSBatR14	Bat parvovirus BS1	5.62	187	1967	13.38	5065	38.80%
S18CXBatR17-2	Bat parvovirus CX1	18.33	719	3531	29.67	5065	69.70%
S18LSBatR61	Bat parvovirus LS1	2.68	73	1012	10.06	5065	20.00%
S17BSBatR05	Bat picornavirus BS1	27.19	431	2922	20.88	7600	38.40%
S17BSBatR11	Bat picornavirus BS2	3.19	127	2369	7.65	7600	31.20%
S18LSBatR79	Bat picornavirus BS2	1.02	54	1624	3.26	7600	21.40%
S18CXBatR05	Bat picornavirus CX1	161.50	4443	7750	81.64	7600	100.00%
S18CXBatR07	Bat picornavirus CX1	235.69	2673	7750	49.28	7600	100.00%
S18CXBatR24	Bat picornavirus CX2	278.85	4304	6878	91.56	7600	90.50%
S18CXBatR07	Bat picornavirus CX2	42.49	720	6414	14.89	7600	84.40%
S18CXBatR17-2	Bat picornavirus CX2	4.77	378	3809	7.66	7600	50.10%
S18CXBatR27	Bat picornavirus CX2	2.85	70	3166	1.48	7600	41.70%
S18CXBatR14-2	Bat picornavirus CX3	48.48	1851	6850	39.30	7600	90.10%
S18CXBatR25	Bat picornavirus CX4	19.40	1586	7253	31.99	7600	95.40%
S18CXBatR30	Bat picornavirus CX4	87.14	579	6831	11.64	7600	89.90%
S18CXBatR09	Bat picornavirus CX5	54.28	2596	6915	53.20	7600	91.00%
S18CXBatR21	Bat picornavirus CX5	9.96	110	3719	2.32	7600	48.90%
S18CXBatR30	Bat picornavirus CX6	36333.15	238682	7829	4448.89	7600	100.00%
S18CXBatR08	Bat picornavirus CX7	1387.89	22863	7715	422.18	7600	100.00%
S18CXBatR11	Bat picornavirus CX7	329.13	6363	7715	120.53	7600	100.00%
S18CXBatR17-2	Bat picornavirus CX7	18.31	707	7449	13.32	7600	98.00%
S18CXBatR28	Bat picornavirus CX7	2.28	166	5112	3.15	7600	67.30%
S18CXBatR17-2	Bat picornavirus CX8	43.23	2850	6683	61.92	7600	87.90%
S18CXBatR17-2	Bat picornavirus CX9	2.83	109	1588	9.99	7600	20.90%
S18LSBatR78	Bat picornavirus LS1	176.93	1324	7025	27.40	7600	92.40%
S16WDBatR60	Bat picornavirus WD1	9.32	357	1621	31.41	7600	21.30%
S18WDBatR02	Bat picornavirus WD2	2.07	64	1159	7.87	7600	15.30%
S17WDBatR07	Bat picornavirus WD3	1.99	111	1540	9.18	7600	20.30%
S15ZKBatR30	Bat picornavirus ZK1	349.97	12405	6914	256.03	7600	91.00%
S15ZKBatR13	Bat picornavirus ZK1	20.92	716	6877	14.59	7600	90.50%
S19MLBatR11	Bat picornavirus ZK1	26.49	1703	6075	34.10	7600	79.90%
S15ZKBatR22	Bat picornavirus ZK1	1.28	78	3055	1.62	7600	40.20%

S18CXBatR14-2	Bat polyomavirus CX1	24.95	966	5129	27.23	5129	100.00%
S15BSBatR04	Bat polyomavirus CX1	5.64	143	4430	3.92	5129	86.40%
S18CXBatR24	Bat rotavirus A type CX1	802895.75	8833939	18249	68532.61	18600	98.10%
S17BSBatR49	Bat rotavirus A type CX1	13602.16	750959	17877	5812.30	18600	96.10%
S17BSBatR35	Bat rotavirus A type CX1	4666.13	361226	17763	2745.89	18600	95.50%
S17BSBatR16	Bat rotavirus A type CX1	321.30	13738	17534	105.40	18600	94.30%
S17BSBatR37	Bat rotavirus A type CX1	66.35	3639	16510	29.21	18600	88.80%
S17BSBatR36	Bat rotavirus A type CX1	39.22	2723	16017	20.98	18600	86.10%
S18LSBatR79	Bat rotavirus A type CX1	74.41	3101	15878	28.12	18600	85.40%
S18LSBatR78	Bat rotavirus A type CX1	39.62	690	14001	5.42	18600	75.30%
S17BSBatR41	Bat rotavirus A type CX1	12.37	930	13840	7.28	18600	74.40%
S18CXBatR23	Bat rotavirus A type CX1	36.27	277	10620	2.15	18600	57.10%
S17BSBatR07	Bat rotavirus A type CX1	5.72	247	8737	1.81	18600	47.00%
S17BSBatR48	Bat rotavirus A type CX1	3.36	126	6798	1.12	18600	36.50%
S17BSBatR12	Bat rotavirus A type CX1	1.19	39	3469	0.60	18600	18.70%
S18CXBatR21	Bat rotavirus A type CX1	3.17	34	2818	0.42	18600	15.20%
S18CXBatR30	Bat rotavirus A type CX1	4.29	28	1876	0.44	18600	10.10%
S18CXBatR29	Bat rotavirus A type CX1	1.22	22	946	0.66	18600	5.10%
S18CXBatR30	Bat RVJ-like rotavirus CX2	1214.49	7986	18293	65.89	18600	98.30%
S18CXBatR21	Bat RVJ-like rotavirus CX2	23.09	266	9445	2.12	18600	50.80%
S18CXBatR10	Bat RVJ-like rotavirus CX2	7.93	246	9217	2.41	18600	49.60%
S18CXBatR16	Bat RVJ-like rotavirus CX2	2.05	118	7241	1.05	18600	38.90%
S18CXBatR13	Bat RVJ-like rotavirus CX2	3.25	46	3289	0.45	18600	17.70%
S18CXBatR23	Bat RVJ-like rotavirus CX2	6.84	54	2999	0.71	18600	16.10%
S18LSBatR79	Bat RVJ-like rotavirus LS1	683.03	16351	18067	148.44	18600	97.10%
S18LSBatR80	Bat RVJ-like rotavirus LS1	15.12	442	13687	4.41	18600	73.60%
S18LSBatR82	Bat RVJ-like rotavirus LS1	4.11	98	5793	1.13	18600	31.10%
S17BSBatR12	Bat RVJ-like rotavirus LS1	3.18	125	5423	1.25	18600	29.20%
S17BSBatR11	Bat RVJ-like rotavirus LS1	1.95	101	4315	1.40	18600	23.20%
S18LSBatR75	Bat RVJ-like rotavirus LS1	1.59	34	2519	0.37	18600	13.50%
S16WDBatR61	Bat rotavirus A type WD1	271.72	9699	17944	62.93	18600	96.50%
S18WDBatR02	Bat rotavirus A type WD1	16.70	578	13517	4.64	18600	72.70%
S17WDBatR01	Bat rotavirus A type WD2	2.22	118	7017	1.03	18600	37.70%
S16WDBatR62	Bat rotavirus A type WD2	1.89	116	6752	1.25	18600	36.30%
S18WDBatR05	Bat rotavirus A type WD2	2.78	108	6513	0.92	18600	35.00%
S18WDBatR08	Bat rotavirus A type WD2	8.46	80	5258	0.59	18600	28.30%
S15ZKBatR13	Bat RVJ-like rotavirus ZK1	261.75	8957	17939	74.54	18600	96.40%
S18LSBatR73	Bat RVJ-like rotavirus ZK1	47.61	793	16788	6.38	18600	90.30%
S15ZKBatR11	Bat RVJ-like rotavirus ZK1	47.17	574	15208	5.49	18600	81.80%

S15ZKBatR27	Bat RVJ-like rotavirus ZK1	3.25	208	10751	1.75	18600	57.80%
S15ZKBatR30	Bat RVJ-like rotavirus ZK1	1.13	40	1852	0.80	18600	10.00%
S16WDBatR58	Bat sapovirus WD1	55.41	2123	7212	41.84	7696	93.70%
S17WDBatR10	Bat sapovirus WD2	178.40	8704	7394	167.20	7696	96.10%
S18WDBatR05	Bat sapovirus WD3	18.30	715	6734	14.74	7696	87.50%
S18WDBatR08	Bat sapovirus WD3	4.35	38	1489	0.79	7696	19.30%
S18CXBatR24	Bat SARS-like coronavirus CX1	32.53	357	13488	1.74	29903	45.10%
S18CXBatR29	Bat SARS-like coronavirus CX1	14.91	272	11110	1.33	29903	37.20%
S18LSBatR79	Bat SARS-like coronavirus LS1	776.17	18255	29756	89.32	29751	100.00%
S18LSBatR61	Bat SARS-like coronavirus LS1	2.00	54	4107	0.26	29751	13.80%
S18LSBatR73	Bat SARS-like coronavirus LS1	0.88	18	1915	0.09	29751	6.40%
S15BSBatR05	Bat tupavirus BS1	32559.39	612185	12187	7179.72	11440	100.00%
S15BSBatR06	Bat tupavirus BS1	2.02	52	2178	0.61	11440	19.00%
S17BSBatR16	Bat tupavirus BS2	2408.63	82448	11950	984.36	11440	100.00%
S18CXBatR13	Bat tupavirus CX1	130.75	1334	5069	38.58	11440	44.30%
S18CXBatR16	Bat tupavirus CX1	1.21	66	2725	1.91	11440	23.80%
S18CXBatR13	Bat tupavirus CX2	155.19	1580	4377	52.92	11440	38.30%
S18CXBatR16	Bat tupavirus CX2	1.06	58	2520	1.93	11440	22.00%
S18CXBatR21	Bat tupavirus CX3	145.79	1581	6276	36.76	11440	54.90%
S18CXBatR09	Bat tupavirus CX3	7.93	377	5679	8.52	11440	49.60%
S18CXBatR23	Bat tupavirus CX3	18.75	138	4688	3.20	11440	41.00%

Supplementary Table 4. Partial Mantel tests showing the effect of host phylogenetic distance, geographic distance and time interval on mammal-associated virome similarity. P values are two-sided and are obtained from 1000 permutations. The precision of permutational P values is 0.001 using 1000 permutations, so the precise values for P < 0.001 are not shown.

Variable	Coefficient of correlation	P value
Phylogenetic distance	0.275	<0.001
Geographic distance	0.078	0.011
Time interval	0.085	0.007

Supplementary Table 5. Partial Mantel tests showing the effect of host phylogenetic distance, geographic distance and time interval on total virome similarity. P values are two-sided and are obtained from 1000 permutations. The precision of permutational P values is 0.001 using 1000 permutations, so the precise values for P < 0.001 are not shown.

Variable	Coefficient of correlation	P value
**Total virome (RNA viruses only) ***		
Phylogenetic distance	0.424	<0.001
Geographic distance	0.353	<0.001
Time interval	0.231	<0.001
**Total virome (grouped by viral Order, DNA and RNA viruses)**		
Phylogenetic distance	0.330	<0.001
Geographic distance	-0.084	0.995
Time interval	0.160	<0.001

*Total virome includes all RNA virus species detected, and this analysis was performed based at the species level. Another analysis was performed by grouping viral species according to their viral order, which include both RNA and DNA viruses. Grouping viruses by order can increase robustness, especially for DNA bacteriophage, as their genome sizes are often very large, of which meta-transcriptomics can only recover a small portion.
