# Supplementary material for: Individual bat virome analysis reveals co-infection and spillover among bats and virus zoonotic potential
Source: Nat Commun. 2023 Jul 10;14:4079. doi: 10.1038/s41467-023-39835-1 (PMC10333379; doi:10.1038/s41467-023-39835-1)
Supplement: Supplementary file 3 — Description of Additional Supplementary Files [file 41467_2023_39835_MOESM3_ESM.pdf]

Description of Additional Supplementary Files

File Name: Supplementary Data 1
Description: Primers used in this study.
