# Supplementary material for: Individual bat virome analysis reveals co-infection and spillover among bats and virus zoonotic potential
Source: Nat Commun. 2023 Jul 10;14:4079. doi: 10.1038/s41467-023-39835-1 (PMC10333379; doi:10.1038/s41467-023-39835-1)
Supplement: Supplementary file 5 — Reporting Summary [file 41467_2023_39835_MOESM5_ESM.pdf]

nature portfolio

Corresponding author(s): Yao-qing Chen, Edward C. Holmes, Yun Feng and Mang Shi

Last updated by author(s): May 26, 2023

Reporting Summary

Nature Portfolio wishes to improve the reproducibility of the work that we publish. This form provides structure for consistency and transparency in reporting. For further information on Nature Portfolio policies, see our Editorial Policies and the Editorial Policy Checklist.

Statistics

For all statistical analyses, confirm that the following items are present in the figure legend, table legend, main text, or Methods section.

n/a	Confirmed	
☐	☒	The exact sample size (*n*) for each experimental group/condition, given as a discrete number and unit of measurement
☐	☒	A statement on whether measurements were taken from distinct samples or whether the same sample was measured repeatedly
☐	☒	The statistical test(s) used AND whether they are one- or two-sided<br>*Only common tests should be described solely by name; describe more complex techniques in the Methods section.*
☐	☒	A description of all covariates tested
☒	☐	A description of any assumptions or corrections, such as tests of normality and adjustment for multiple comparisons
☐	☒	A full description of the statistical parameters including central tendency (e.g. means) or other basic estimates (e.g. regression coefficient) AND variation (e.g. standard deviation) or associated estimates of uncertainty (e.g. confidence intervals)
☐	☒	For null hypothesis testing, the test statistic (e.g. *F*, *t*, *r*) with confidence intervals, effect sizes, degrees of freedom and *P* value noted<br>*Give P values as exact values whenever suitable.*
☒	☐	For Bayesian analysis, information on the choice of priors and Markov chain Monte Carlo settings
☒	☐	For hierarchical and complex designs, identification of the appropriate level for tests and full reporting of outcomes
☐	☒	Estimates of effect sizes (e.g. Cohen's *d*, Pearson's *r*), indicating how they were calculated

Our web collection on statistics for biologists contains articles on many of the points above.

Software and code

Policy information about availability of computer code

Data collection	No software was used for data collection.
Data analysis	Bowtie 2 version 2.4.1; MEGAHIT 1.2.8; Diamond 0.9.25; SeqMan 7.1; NCBI ORFfinder 0.4.3; MAFFT 7.48; PhyML 3.0; Simplot 3.5.1; MODELLER 10.3; GROMACS 2022.3; PLUMED 2.7.4; FoldX version 4; PyMOL version 2.4.2; R 4.2.0; igraph (R package) 1.3.1; ecodist (R package) 2.0.9; vegan (R package) 2.6-2; tidyverse (R package) 1.3.1; ComplexHeatmap (R package) 2.12.0; RColorBrewer (R package) 1.1-3; circlize (R package) 0.4.14; SoDA (R package) 1.0-6.1; ape (R package) 5.6-2; open source code: https://github.com/maxbonomi/bat-MD; custom codes: https://github.com/Augustpan/Individual-Bat-Virome

For manuscripts utilizing custom algorithms or software that are central to the research but not yet described in published literature, software must be made available to editors and reviewers. We strongly encourage code deposition in a community repository (e.g. GitHub). See the Nature Portfolio guidelines for submitting code & software for further information.

Data

Policy information about availability of data

All manuscripts must include a data availability statement. This statement should provide the following information, where applicable:

- Accession codes, unique identifiers, or web links for publicly available datasets
- A description of any restrictions on data availability
- For clinical datasets or third party data, please ensure that the statement adheres to our policy

All necessary codes and raw data to reproduce our results are available at a public github repository: https://github.com/Augustpan/Individual-Bat-Virome. Raw meta-transcriptomic sequences have been deposited in the NCBI SRA database, and all assembled genomes have been deposited in the NCBI GenBank database. Accession numbers to these data have been made public upon publication.

Research involving human participants, their data, or biological material

Policy information about studies with human participants or human data. See also policy information about sex, gender (identity/presentation), and sexual orientation and race, ethnicity and racism.

Reporting on sex and gender	N/A
Reporting on race, ethnicity, or other socially relevant groupings	N/A
Population characteristics	N/A
Recruitment	N/A
Ethics oversight	N/A

Note that full information on the approval of the study protocol must also be provided in the manuscript.

Field-specific reporting

Please select the one below that is the best fit for your research. If you are not sure, read the appropriate sections before making your selection.

☒ Life sciences ☐ Behavioural & social sciences ☐ Ecological, evolutionary & environmental sciences

For a reference copy of the document with all sections, see nature.com/documents/nr-reporting-summary-flat.pdf

Life sciences study design

All studies must disclose on these points even when the disclosure is negative.

Sample size	149 individuals of bat were captured and used for analysis. Sample size for samples from covalescent/vaccinated individuals was chosen according to or exceeding standards in the field, and in most cases exceeded 10 samples per group. The number of bat individual for dominant bat genera was at least 20, and this guaranteed enough sample size for viral diversity comparison and cross-species transmission analysis in our major results.
Data exclusions	No data exclusions.
Replication	All raw meta-transcriptomic sequences and assembled genomes are publicly available at NCBI databases, so that anyone can reanalyze these data. And we also provided detailed methods, codes and necessary data to fully reproduce our analysis.
Randomization	All samples were randomly allocated into different groups according their biological characteristics, such as species.
Blinding	All data were analyzed blindingly without grouping factors informed, only statistical comparison were taken according to grouping information.

Reporting for specific materials, systems and methods

We require information from authors about some types of materials, experimental systems and methods used in many studies. Here, indicate whether each material, system or method listed is relevant to your study. If you are not sure if a list item applies to your research, read the appropriate section before selecting a response.

Materials & experimental systems

n/a	Involved in the study
☐	☒ Antibodies
☐	☒ Eukaryotic cell lines
☒	☐ Palaeontology and archaeology
☐	☒ Animals and other organisms
☒	☐ Clinical data
☒	☐ Dual use research of concern
☒	☐ Plants

Methods

n/a	Involved in the study
☒	☐ ChIP-seq
☒	☐ Flow cytometry
☒	☐ MRI-based neuroimaging

Antibodies

Antibodies used	Goat Anti-Human IgG conjugate with HRP used in ELISA were produced by Jackson ImmunoResearch (catalog number: #109-035-098).
Validation	Based on immunoelectrophoresis and/or ELISA, the antibody reacts with the Fc portion of human IgG heavy chain but not with the Fab portion of human IgG. No antibody was detected against human IgM or IgA, or against non-immunoglobulin serum proteins. The antibody has been tested by ELISA and/or solid phase adsorbed to ensure minimal cross-reaction with bovine, horse, and mouse serum proteins, but it may cross-react with immunoglobulins from other species.

Eukaryotic cell lines

Policy information about cell lines and Sex and Gender in Research

Cell line source(s)	The cell line used in this study (HEK293T) was obtained from American Type Culture Collection (ATCC, catalog number: CRL-3216).
Authentication	The cell line was authenticated using Short Tandem Repeat (STR) makers. We compared the nine STR makers that are widely used for identifying HEK293T, which are Amelogenin, CSF1PO, D13S317, D16S539, D5S818, D7S820, THO1, TPOX and vWA. The STR profile supported that the cells used in this study was indeed HEK293T.
Mycoplasma contamination	The cell line was test negative for mycoplasma.
Commonly misidentified lines (See ICLAC register)	The most commonly misidentified cell line is HEK293. Our STR profiling indicate that the cells we used in this study is indeed HEK293T instead of HEK293.

Animals and other research organisms

Policy information about studies involving animals; ARRIVE guidelines recommended for reporting animal research, and Sex and Gender in Research

Laboratory animals	Laboratory animals not involved.
Wild animals	Live bat individuals of 15 species (mainly Aselliscus stoliczkanus, Rousettus leschenaultii, and Rhinolophus pusillus) from six bat genera were captured using nets near their natural habitats, primarily caves. The captured individuals were immediately transported to the laboratory by placing them in cages or bags inside a vehicle. Subsequently, they were anesthetized and dissected. Organ and tissue samples were then stored at -80 degrees Celsius.
Reporting on sex	Sex-based analysis not included.
Field-collected samples	Bat individuals were collected from six different counties within Yunnan Province, China. The housing environment for the collected bat individuals was suitable, regularly maintained, with an appropriate temperature range and a proper photoperiod. The field-collected bat samples were handled according to appropriate end-of-experiment-protocols and ethical requirements.
Ethics oversight	This research, including the procedures and protocols of specimen collection and processing was reviewed by the Medical Ethics Committee of the Yunnan Institute of Endemic Diseases Control and Prevention (No. 20160002).

Note that full information on the approval of the study protocol must also be provided in the manuscript.
